# EEG Evidence of Altered Functional Connectivity and Microstate in Children Orphaned by HIV/AIDS

**DOI:** 10.3389/fpsyt.2022.898716

**Published:** 2022-06-29

**Authors:** Huang Gu, Xueke Shan, Hui He, Junfeng Zhao, Xiaoming Li

**Affiliations:** ^1^Institute of Behavior and Psychology, School of Psychology, Henan University, Kaifeng, China; ^2^Department of Health Promotion, Education, and Behavior, University of South Carolina, Columbia, SC, United States

**Keywords:** children orphaned by HIV/AIDS, early life stress, resting-state, microstate, functional connectivity

## Abstract

Children orphaned by HIV/AIDS (“AIDS orphans”) suffer numerous early-life adverse events which have a long-lasting effect on brain function. Although previous studies found altered electroencephalography (EEG) oscillation during resting state in children orphaned by HIV/AIDS, data are limited regarding the alterations in connectivity and microstate. The current study aimed to investigate the functional connectivity (FC) and microstate in children orphaned by HIV/AIDS with resting-state EEG data. Data were recorded from 63 children orphaned by HIV/AIDS and 65 non-orphan controls during a close-eyes resting state. The differences in phase-locking value (PLV) of global average FC and temporal dynamics of microstate were compared between groups. For functional connectivity, children orphaned by HIV/AIDS showed decreased connectivity in alpha, beta, theta, and delta band compared with non-orphan controls. For microstate, EEG results demonstrated that children orphaned by HIV/AIDS show increased duration and coverage of microstate C, decreased occurrence and coverage of microstate B, and decreased occurrence of microstate D than non-orphan controls. These findings suggest that the microstate and functional connectivity has altered in children orphaned by HIV/AIDS compared with non-orphan controls and provide additional evidence that early life stress (ELS) would alter the structure and function of the brain and increase the risk of psychiatric disorders.

## Introduction

Children orphaned by HIV/AIDS (“AIDS orphans”) were defined as children under the age of 18 years who had lost one or both parents to HIV-related illnesses (1). The United Nations International Children's Emergency Fund estimated that there were 15.4 million AIDS orphans worldwide by 2021 (2). The number of AIDS orphans could have reached 260,000–400,000 in China (3) with an increasing trend. When they grow up, AIDS orphans may suffer numerous early life stress (ELS) events, such as parental death, poverty, disrupted school attendance, and stigma. According to previous studies, these ELS events have been associated with changes in brain structure and function (4–8). Understanding these changes promises fundamental insights into the underlying pathophysiology and may eventually help establish a much sought-after biomarker of ELS.

Brain function development after ELS has mostly been assessed using functional magnetic resonance imaging (fMRI). By assessing brain activity and connectivity, recent fMRI studies have found a reduction in the volume of the hippocampus, prefrontal cortex (PFC), and corpus callosum in children with ELS (9, 10). Besides the brain structure, the alterations in brain functional connectivity (FC) have also been found, such as increased connectivity between the ventral striatum and lateral PFC (11, 12), and decreased amygdala-PFC connectivity (13, 14), or reduced ventral tegmental area-hippocampal connectivity (15). While findings from fMRI studies are all in low frequencies, there is still a lack of knowledge about brain function in a resting state at higher frequencies. To address this, electroencephalography (EEG) can provide a new perspective because of its higher temporal resolution. Mounting evidence indicates that resting-state EEG activity is related to brain functions (16–18). For example, in the attentional function, alpha oscillations were considered to clear sensory information from distractors (19). The theta/beta ratio had a negative correlation with information processing speed and attention performance (20). Therefore, analysis of resting-state EEG characteristics may reveal the alteration of brain functions in AIDS orphans.

In the commonly resting EEG analysis, a promising approach is a microstate. EEG microstates are defined as global patterns of scalp potential topographies which remain stable for a certain period of time (50–100 ms) before rapidly transitioning to different microstates (21). Most studies demonstrate that the same four classes of archetypal microstates which were labeled as A, B, C, and D can explain most of the global topographic variance (22). According to fMRI-EEG studies, different microstates correspond to certain specific resting-state functional networks. Specifically, microstate class A was associated with the auditory processing, microstate class B with the visual network, microstate class C with the salience network (SN), and microstate class D with the attention (23, 24). According to previous studies, the temporal parameters, such as duration (the mean duration of a microstate class in milliseconds), occurrence (the mean frequency of observation of a microstate class per second), and coverage (the proportion of the total time spent in a microstate class) could reflect the function of brain networks and these parameters could be altered by age, pressure, and diseases (22, 25, 26).

Following what was previously reported, there are many techniques to estimate resting-state EEG FC (27, 28). Among these techniques, The phase locking value (PLV) is especially suitable for connectivity analysis because it quantifies coupling between pairs of electrodes and measures the synchronization of temporal relationships of neural signals independent of their signal amplitude (29, 30). PLV has been used in previous studies to examine FC (31). For instance, a study that measures FC between default mode networks regions of interest and the medial prefrontal regions using PLV found decreased connectivity in the alpha band in older people (32). Rogala et al. found a positive correlation between resting-state PLV and the power of the beta-2 band (22–29 Hz), demonstrating that beta band activity plays an important role in the attentional process (16). In addition, recent studies investigated the correlation between EEG FC and fMRI FC by using different techniques and found that PLV is significantly correlated with fMRI networks compared with other FC methods (33).

This study aimed to investigate the large-scale network across the whole brain in AIDS orphans and compared it to non-orphan controls. To achieve this, the present research will use FC and the microstate approach to analyze the EEG data. The temporal parameters (duration, occurrence, and coverage) will be assessed for the microstate. For FC, the PLV will be used to calculate the functional connection.

## Method

### Participants

Data were derived from a larger neurodevelopmental study in which a total of 91 AIDS orphans and 66 non-orphan children (controls) were recruited from the local communities and school systems in central rural China. The study was open to children at 8–18 years of age who did not have HIV/AIDS-related illnesses. Age eligibility was verified through the local community leaders, school records, or caregivers. Among these participants, 65 AIDS orphans and 66 controls completed the EEG experiment. All the subjects had a normal or adequately corrected vision, were right-handed, and reported no history of mental, medical, or neurological disorders. At the end of the experiment, they received an age-appropriate gift as a token of appreciation. Written informed consent was obtained for the study. Two AIDS orphans and one control child were excluded from further analysis due to an unfinished EEG experiment. The study protocol was approved by the Institutional Review Boards at the University of South Carolina in the United States and Henan University in China (IRB 00007212).

### Measures and Procedures

#### EEG Recording and Preprocessing

**Figure 1:**
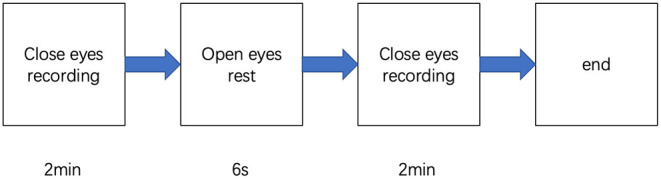
Procedures of the experiment.

**Figure 2:**
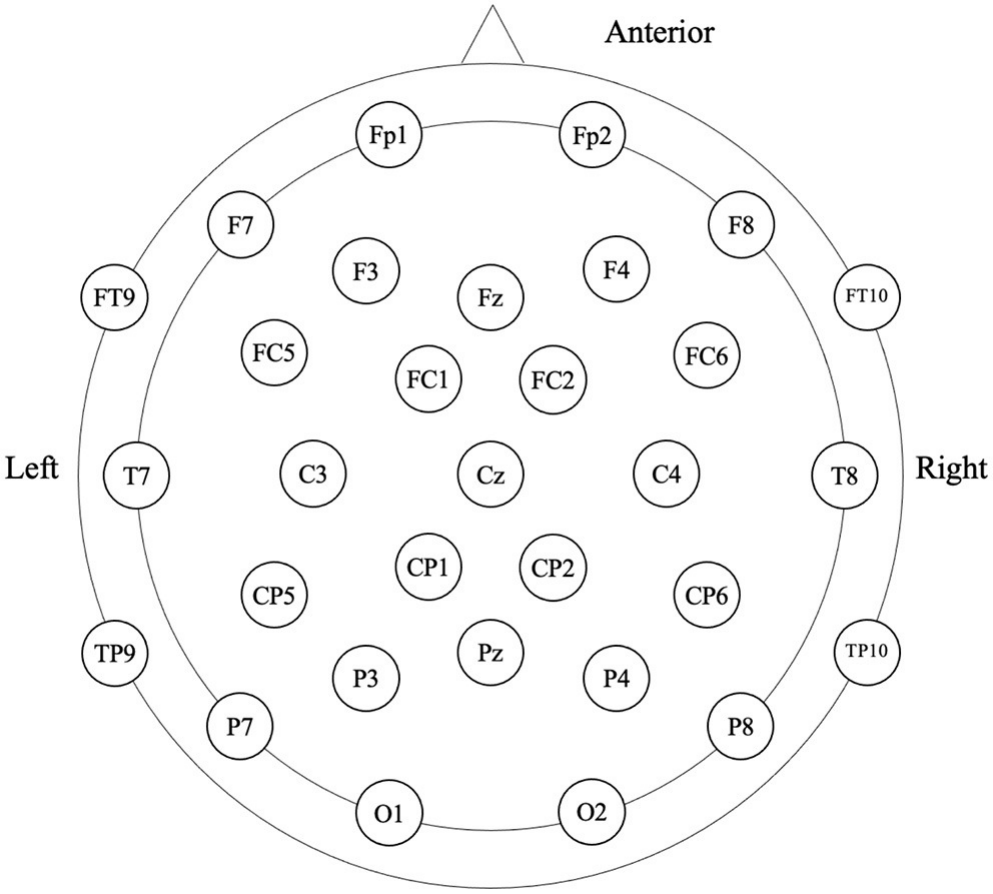
Electroencephalography (EEG) cap configuration with international 10–20 system.

Participants were in the eyes-closed resting state when 4-min spontaneous EEG data were collected (Figure 1). The EEG was recorded from a 32-scalp standard channel cap (10/20 system; Brain Products, Munich, Germany) (Figure 2). An electrooculogram (EOG) was recorded from electrodes placed at the outer canthi of the right eye. All electrode recordings were online referenced to FCz. All inter-electrode impedances were maintained below 5 kΩ. The EEG and EOG signals were amplified using a 0.01–100 Hz bandpass filter and continuously sampled at 500 Hz/channel for offline analysis.

After data acquisition, offline preprocessing was performed with EEGLAB (34). The EEG data were re-referenced to the common average reference. Then, the data were corrected for artifacts using Independent Component Analysis (ICA). Subsequently, all data were bandpass filtered at 2–20 Hz and segmented in 2 s epochs. Segments were rejected from further analyses if amplitudes exceeded ±100 uV.

#### EEG Connectivity Analysis

As a measure of synchrony, PLV is used as an indicator of FC between different brain regions. Compared with other indicators of FC, PLV does not depend on the spectral power of recorded signals and is more sensitive for measuring FC (16). In addition, it has good reliability for all frequency bands.

The current source density (CSD) method was used to transform EEG data from scalp electrode space into a reference-free montage (35). The EEG signals of all electrodes from CSD-converted montage are band-pass filtered into four frequency bands [delta (2–4 Hz), theta (4–8 Hz), alpha (8–13 Hz), and beta (13–20 Hz)] and transformed into analytical EEG signals using Hilbert transform (29). For each frequency band, the PLVs were calculated for all pairs of electrodes and generated an *N* × *N* synchronization matrix with *N* equal to 29, in which each entry *N*_*i,j*_ contains the value of the PLV for the channels *i* and *j*. The global mean PLV of each subject was calculated based on the *N* × *N* matrix.

### Microstate Analysis

As a method of studying EEG, microstate analysis regards the EEG signal as a series of quasi-stable microstates and access the global functional state of the brain by comparing the characteristic of microstate time series (23).

Microstate analysis was performed with the microstate analysis plugin (Version 1.2; http://www.thomaskoenig.ch/Download/EEGLAB_Microstates/) for EEGLAB in Matlab 2018b. The steps were as follows. First, 1,000 global field power (GFP) peaks were selected randomly and were submitted to Atomize-Agglomerate Hierarchical Clustering (AAHC) analysis. Next for each cluster number of microstate maps from 3 to 6 was determined. According to the cross-validation criterion, we found four microstates could explain the variance of 76.26 and 71.75% for two groups. Then, a similar clustering analysis was performed at the group level based on the microstate template maps of all the participants. For the statistical analysis, the three temporal features of the microstates were extracted (duration, occurrence, and, coverage).

### Statistical Analysis

In this study, the *t*-test was used to compare the demographic variables between two groups. All the variables with a significant difference will be used as covariates in all the subsequent analyses.

The group differences of EEG connectivity analysis were evaluated separately on each frequency band. A comparison of the group mean PLVs was conducted using one-way analysis of variance (ANOVA). For microstate analysis, the repeated-measures ANOVA was applied with microstate class (A, B, C, and D) as a within-subject factor, and group (AIDS orphans and controls) as a between-subject factor. One-way ANOVA was used to compare groups for temporal parameters of each microstate when the main effects or interactions were significant.

A greenhouse-Geisser correction was conducted to adjust *p*-values when appropriate. All analyses were calculated by SPSS 25.0.

## Results

In demographic variables, age was found to be a significant difference between the two groups and included as a covariate in subsequent analyses.

### Global Connectivity

**Figure 3:**
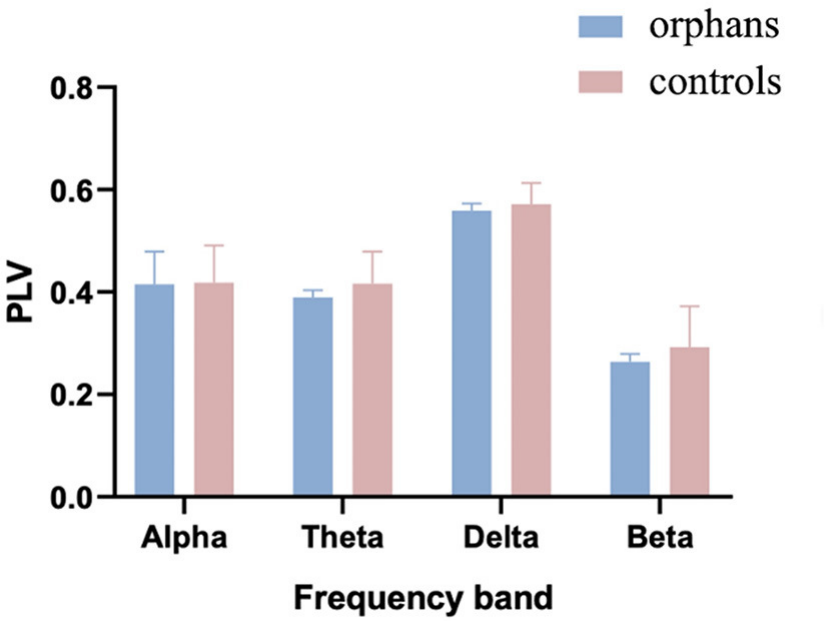
The mean phase-locking value (PLV) averaged over all pairs of EEG channels for AIDS orphans and controls in four frequency bands.

The group difference in mean global PLV was calculated with one-way ANOVA and age as a covariate. As shown in Figure 3, the mean PLV was significantly lower in AIDS orphans (alpha: 0.415 ± 0.064; theta: 0.389 ± 0.014; delta: 0.559 ± 0.013; and beta: 0.263 ± 0.015) than controls (alpha: 0.419 ±0.073; theta: 0.416 ± 0.062; delta: 0.0571 ± 0.410; and beta: 0.293 ± 0.080) in all frequency bands (alpha: *F*_(1, 128)_ = 8.446, *p* = 0.004, *η*^2^ = 0.062; theta: *F*_(1, 128)_ = 24.337, *p* < 0.001, *η*^2^ = 0.160; delta: *F*_(1, 128)_ = 15.395, *p* < 0.001, *η*^2^ = 0.107; and beta: *F*_(1, 128)_ = 22.989, *p* < 0.001, *η*^2^ = 0.152) (Table 1).

**Table 1:** The mean phase-locking value (PLV) for AIDS orphans and controls at each frequency.

	**Alpha**	**Theta**	**Delta**	**Beta**
orphans	0.415 ± 0.064	0.389 ± 0.014	0.559 ± 0.013	0.263 ± 0.015
controls	0.419 ± 0.073	0.416 ± 0.062	0.0571 ± 0.410	0.293 ± 0.080
F	8.446	24.337	15.395	22.989
*p*	**0.004**	**<** **0.001**	**<** **0.001**	**<** **0.001**

*Values are expressed as means ± SD; analysis of variance (ANOVA) test followed by Greenhouse-Geisser correction*.

*Significant differences are marked in bold*.

*Orphans, AIDS orphans*.

### Microstate Results

**Figure 4:**
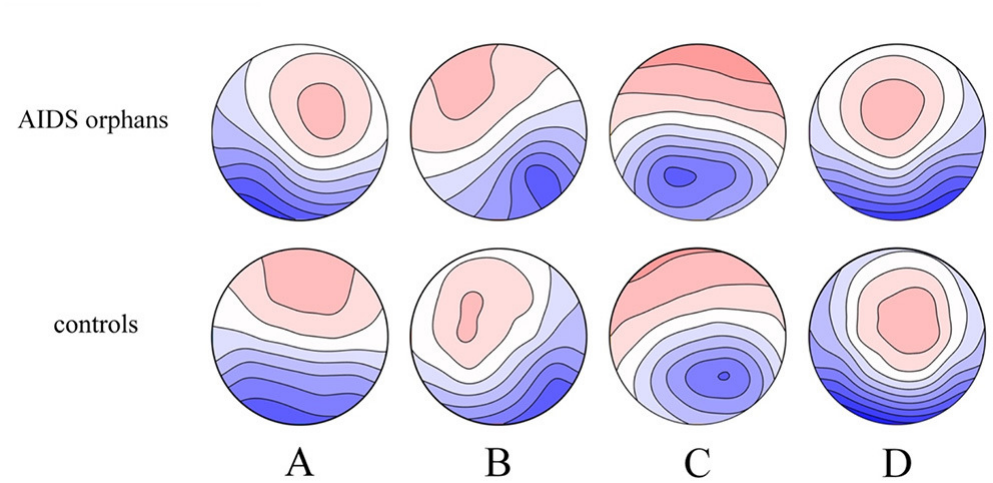
Spatial configuration of the four microstate classes. Each row displays the four topographic configurations **(A–D)** for each group. AIDS orphans, controls.

The four microstate classes (A, B, C, and D) of orphans and controls obtained in the whole groups had topographies comparable with those previously found in most microstate studies (Figure 4). These microstates accounted for an average of 76.26% (SD = 5.4%) and 71.75% (SD = 7.6%) of the global variance in the AIDS orphans and control group, respectively.

We found significant class (A, B, C, and D) × group (AIDS orphans and controls) interactions for coverage (*F*_(3, 375)_ = 3.492, *p* = 0.020, $\eta_{p}^{2}$ = 0.027), and occurrence (*F*_(3, 375)_ = 5.756, *p* = 0.001, $\eta_{p}^{2}$ = 0.044). In addition, the main effect of duration (*F*_(1, 125)_ = 4.510, *p* = 0.036, $\eta_{p}^{2}$ = 0.035) was significant (Table 2).

**Table 2:** The results of the 2-way ANOVA for duration, occurrence, and coverage.

	**F(df)**		***p***	*** $\eta_{p}^{2}$ ***
**Duration**				
Main effects				
Class	F_(3, 375)_	0.424	0.709	0.003
**Group**	F_(1, 125)_	4.51	**0.036**	0.035
Age	F_(1, 125)_	9.792	**0.002**	0.073
2-way interaction				
Class * group	F_(3, 375)_	2.25	0.091	0.018
Class * age	F_(3, 375)_	0.181	0.886	0.001
**Occurrence**				
Main effects				
Class	F_(3, 375)_	0.963	0.403	0.008
Group	F_(1, 125)_	3.243	0.074	0.025
Age	F_(1, 125)_	10.549	**0.001**	0.078
2-way interaction				
**Class** *group	F_(3, 375)_	5.756	**0.001**	0.044
Class * age	F_(3, 375)_	1.127	0.335	0.009
**Coverage**				
Main effects				
Class	F_(3, 375)_	0.616	0.605	0.005
Group	F_(1, 125)_	1.609	0.207	0.013
Age	F_(1, 125)_	2.685	0.104	0.021
2-way interaction				
**Class** *group	F_(3, 375)_	3.492	**0.020**	0.027
Class * age	F_(3, 375)_	0.504	0.658	0.004

*F(df), F-test (degrees of freedom); p, p-value; $\eta_{p}^{2}$, partial eta square*.

*Significant results and differences are marked in bold*.

*Class, microstate class*.

The group differences were found in specific microstate classes. Specifically, for microstate coverage, orphans showed a significant decrease of microstate B (*F*_(1, 125)_ = 4.234, *p* = 0.042, *η*^2^ = 0.033), and a significant increase of microstate C (*F*_(1, 125)_ = 6.227, *p* = 0.014, *η*^2^ = 0.047) than controls. The occurrences of microstate B (*F*_(1, 125)_ = 8.262, *p* = 0.005, *η*^2^ = 0.062) and D (*F*_(1, 125)_ = 8.613, *p* = 0.004, *η*^2^ = 0.064) were higher in controls than in AIDS orphans. AIDS orphans showed significantly increased microstate C duration (*F*_(1, 125)_ = 8.028, *p* = 0.005, *η*^2^ = 0.060) compared with controls (Table 3).

**Table 3:** The mean for all microstate parameters of AIDS orphans and controls.

		**Microstate A**	**Microstate B**	**Microstate C**	**Microstate D**
Duration (ms)	orphans	67.13 ± 9.95	67.38 ± 10.98	68.65 ± 13.71	64.22 ± 11.59
	controls	67.93 ± 10.57	70.19 ± 10.65	63.95 ± 10.33	64.10 ± 7.98
	F (*p*)	1.896 (0.171)	0.028 (0.869)	8.028 (**0.005**)	1.562 (0.214)
Occurrence (s)	orphans	3.78 ± 0.64	3.78 ± 0.73	3.97 ± 0.79	3.77 ± 0.68
	controls	3.66 ± 0.59	4.03 ± 0.65	3.61 ± 0.69	3.90 ± 0.78
	F (*p*)	0.002 (0.964)	8.262 (**0.005**)	1.365 (0.245)	8.613 (**0.004**)
Coverage (%)	orphans	24.76 ± 4.81	24.98 ± 5.54	26.46 ± 6.63	23.81 ± 5.82
	controls	24.58 ± 5.75	27.71 ± 5.65	23.02 ± 6.29	24.69 ± 5.84
	F (*p*)	0.432 (0.512)	4.234 (0.042)	6.227 (0.014)	1.952 (0.165)

*Values are expressed as means ± SD; analysis of variance (ANOVA) test followed by Greenhouse-Geisser correction*.

*Significant differences are marked in bold*.

*Orphans, AIDS orphans*.

## Discussion

The present study aimed to investigate the difference in brain function between AIDS orphans and controls from the perspective of whole brain activities. Here, two novel analytical approaches were used to extract the information from the resting-state EEG data. First, microstate analysis evaluated the spontaneous brain activity and temporal dynamics resting-state networks (RSNs). Second, the altered FC of large-scale brain networks in AIDS orphans was measured. The result of this study showed alterations in microstate parameters and lower FC in all frequency bands for AIDS orphans. These results provide new insight into the brain development of AIDS orphans.

The results of FC suggest that the brain structure and function, as well as development, can be altered even damaged by ELS. This finding is consistent with previous studies that demonstrated ELS may have a negative effect on brain. A large body of studies has highlighted the impaired cognitive and affective functioning in children who experienced ELS (36–38). Wang et al. (39)found a decrease FC within prefrontal-limbic-thalamic-cerebella in major depressive disorder patients with ELS. In a study of adolescents with post-traumatic stress disorder, decreased connectivity between the amygdala and mPFC was observed compared with controls (40). In addition, the current study investigated the difference of functional networks between AIDS orphans and controls in four frequency bands. Oscillations in different frequency bands are often related to cognitive functions. The relationship between alpha oscillation and alertness has been reported in several studies (41, 42). Theta oscillation play an important role in working memory (43). Activity of the delta band was observed during the feedback and oscillation of beta related with sensorimotor decision-making (44–48). Thus, the decrease of FC in all frequency bands indicates that the defects in the brain function of AIDS orphans.

In this study, we found an increase in duration and coverage of microstate C in AIDS orphans compared with controls. This result is consistent with previous studies which found the sensitivity to perception altered (49) and neural responses to salient stimuli enhanced in the ELS sample (50). These neural responses are included in SN, which correspond to microstate C. Thus, the increase of microstate C may represent the individual becoming more sensitive to salience events (51). In addition, the SN connectivity in insula was found to be increased in trauma-exposed youth (52) and the SN alteration was found in patients with major depressive disorder (53), posttraumatic stress disorder (54), and anxiety disorders (55).

In contrast to microstate C, we found a reduction in occurrence and coverage of microstate B and a reduction in the occurrence of microstate D in AIDS orphans. According to previous studies, microstate B and D are related to the visual network and attention network, respectively. The decrease in microstate may represent a deficit in attention of AIDS orphans (56–58), which is consistent with the results of FC. Similar results were reported in patients with psychiatric disorders. For instance, studies with schizophrenic found the reduction in microstate B and D (56, 59, 60). A study on bipolar disorder showed that patients with bipolar disorder have a significant reduction in microstate B (61). In combination with the result of microstate C, this study provided further evidence that individuals who experience ELS are more likely to develop psychiatric disorders. Therefore, the altered microstate in AIDS orphans may be a predictor of mental illness.

It is possible that our results reflect impaired brain function in AIDS orphans. These findings give further support to the diatheses-stress hypotheses that the brain adapts to ELS by releasing mediators which may provoke dendritic stunting and atrophy (62, 63) and consequently affect the structure and function of the brain. In addition, previous studies on the effect of ELS on attention were based on task or certain regionals (36, 64). However, in this study, two methods based on the large-scale resting-state EEG data analysis found defects in attention function, which provided further evidence that ELS has effect on attention.

## Conclusion

The present study showed decreased FC and different microstate dynamics in AIDS orphans. With two independent approaches to analyze EEG resting-state data, we found alterations in the brain function in AIDS orphans, and those alterations were likely to be caused by ELS. These results suggest that functional imaging may be used to detect latent neurodevelopmental effects of ELS exposure, facilitating a better understanding of the pathophysiology and treatment of ELS-related conditions.

### Limitations and Future Directions

One limitation of this study is that we only explore the whole-brain network from two different perspectives. Other methods, such as graph theory as well as long- and short-distance FC, have been used to analyze FC in recent studies. Hence, these methods will be taken into account to investigate the large-scale brain network in further studies. In addition, the research is a cross-sectional study. According to previous studies, ELS has a sustained and life-long impact on the brain. The results of this study reveal the development trajectory of brain with individuals who are preadolescents and undergoing ELS. Thus, the developmental trajectory throughout puberty needs to be explored in a longitudinal study in future studies. Furthermore, this study only explored the characteristics of large-scale resting-state EEG and found the effect of ELS on brain function. However, the effect of ELS on specific cognitive function and its potential neural circuit have not been explored and analyzed. Therefore, in future studies, we will focus on the role of the ventral prefrontal cortex in the acquisition of threat conditions in individuals who experience ELS and exploring its subregional contributions to fear learning and extinction.

## Data Availability Statement

The datasets presented in this article are not readily available due to privacy or ethical restrictions. Requests to access the datasets should be directed to JZ, jfzhao63@hotmail.com.

## Ethics Statement

The study protocol was approved by the Institutional Review Boards at University of South Carolina in the United States and Henan University in China. Written informed consent to participate in this study was provided by the participants' legal guardian.

## Author Contributions

HG, XS, and HH designed the study and drafted the manuscript. XS and HH performed the study, analyzed the data, and editing of the paper. JZ and XL revised the paper. All authors contributed to the article and approved the submitted version.

## Funding

This work was supported by the National Social Science Foundation of China, NSSFC (Grant Number 19BSH111), the Social Science Planning Project of Henan Province (2021CJY051), the Humanities and Social Science Research Project of Henan Provincial Department of Education grant number (2020-ZDJH-026), the Science and Technology Research Project of Henan Provincial Department of Science and Technology (Grant Number 212102310985), and the Henan University Philosophy and Social Science Innovation Team (2019CXTD009).

## Conflict of Interest

The authors declare that the research was conducted in the absence of any commercial or financial relationships that could be construed as a potential conflict of interest.

## Publisher's Note

All claims expressed in this article are solely those of the authors and do not necessarily represent those of their affiliated organizations, or those of the publisher, the editors and the reviewers. Any product that may be evaluated in this article, or claim that may be made by its manufacturer, is not guaranteed or endorsed by the publisher.
